# Cell-Specific Vulnerability of Human Glioblastoma and Astrocytoma Cells to Mephedrone—An In Vitro Study

**DOI:** 10.3390/molecules30112277

**Published:** 2025-05-22

**Authors:** Marta Marszalek-Grabska, Marta Kinga Lemieszek, Michal Chojnacki, Sylwia Winiarczyk, Joanna Jakubowicz-Gil, Barbara Zarzyka, Jarosław Pawelec, Jolanta H. Kotlinska, Wojciech Rzeski, Waldemar A. Turski

**Affiliations:** 1 Department of Experimental and Clinical Pharmacology, Medical University, Jaczewskiego 8b, 20-090 Lublin, Poland; marta.marszalek-grabska@umlub.pl; 2 Department of Medical Biology, Institute of Rural Health, Jaczewskiego 2, 20-090 Lublin, Polandchojnacki.michal@imw.lublin.pl (M.C.);; 3 Department of Functional Anatomy and Cytobiology, Institute of Biological Sciences, Maria Curie-Skłodowska University, Akademicka 19, 20-033 Lublin, Poland; 4 Institute Microscope Laboratory, Institute of Biological Sciences, Maria Curie-Skłodowska University, Akademicka 19, 20-033 Lublin, Poland; 5 Department of Pharmacology and Pharmacodynamics, Medical University, Chodźki 4a, 20-093 Lublin, Poland; 6 Department of Neurotoxicology, Institute of Rural Health, Jaczewskiego 2, 20-090 Lublin, Poland

**Keywords:** mephedrone, glioblastoma, cell lines, proliferation, vacuolization

## Abstract

Glioblastoma multiforme is a highly aggressive intrinsic brain tumor with a very poor survival rate. The main treatment for cancer is surgery combined with postoperative radiotherapy and temozolomide chemotherapy. Since the outcomes of treatment are unsatisfactory, the search for more effective drugs is crucial. Our previous study indicated that mephedrone, a synthetic cathinone, reduced neuron and astrocyte viability and oligodendrocyte proliferation. The aim of the present study was to investigate the effect of mephedrone on selected human glioblastoma (LN-18, LN-229, T98G) and human anaplastic astrocytoma (MOGGCCM) cell lines. The effects of mephedrone on cell viability and proliferation, DNA synthesis, cell cycle progression and the type of cell death were studied. Our results showed that mephedrone possesses potential anticancer activity. The viability and proliferation of all four human glioblastoma and human anaplastic astrocytoma cell lines used were decreased in a concentration-dependent manner. Studies conducted on LN-18 and T98G cells confirmed the significant antiproliferative properties of mephedrone, which reduced DNA synthesis and affected cell cycle progression. Microscopic evaluation supported the antiproliferative effect of the tested compounds. Moreover, substantial cytoplasmic vacuolization in the LN-18 cell line was revealed. This finding may indicate the potential of mephedrone in anticancer therapy.

## 1. Introduction

General pharmacotherapy for cancer involves the induction of caspase-dependent apoptotic cell death through mechanisms such as DNA damage [1], disruptions of the cytoskeleton [2], or the induction of endoplasmic reticulum stress [3]. Many cancer cells, however, harbor mutations in tumor suppressor genes that control programmed cell death, making them relatively resistant to this type of cell death. Additionally, tumor cells that initially respond to standard chemotherapy often develop drug resistance over time due to the intensification of processes for removing the drug from the cell or DNA repair capacity [4].

Glioblastoma multiforme is the most prevalent type of primary brain tumor, representing around 40% of all intracranial tumors [5]. It is an extremely aggressive and persistent hypoxic tumor, with most patients surviving less than two years after their initial diagnosis.

Currently, the main treatment is surgery combined with postoperative radiotherapy and chemotherapy with temozolomide [6]. Temozolomide is an orally bioavailable alkylating agent able to penetrate the blood–brain barrier and modify DNA or RNA [7]. Temozolomide, however, has adverse side effects due to its high toxicity and the development of resistance by tumor tissue over time [8]. To improve pharmacotherapy for gliomas, immunotherapy, targeted therapy, and other new treatment options have been designed, but the overall outcome remains unsatisfactory [9]. Therefore, there is a need to create novel therapeutic strategies or identify effective antitumor agents that can improve the prognosis of gliomas.

Currently, alternative cell death pathways based on nonapoptotic mechanisms are being investigated in order to improve cancer pharmacotherapy. These include specialized forms of necrosis, e.g., oncosis, necroptosis, and paraptosis [10], as well as death associated with the accumulation of autophagosomes [11]. Among them, methuosis has also attracted a lot of attention. Methuosis—from the Greek *methuo*, to drink to intoxication—is a unique type of nonapoptotic cell death [12]. It triggers macropinocytosis, along with disruptions in clathrin-independent endocytic vesicle transport, leading to the buildup of large vacuoles that disrupt the integrity of the cellular membrane [13]. The first data on the molecular mechanisms of methuosis were derived from studies on mutation profiles in glioblastoma multiforme, where this form of cell death was induced by the ectopic expression of activated Ras and Rac GTPases [12]. However, the potential of using this unconventional cell death pathway to target apoptosis-resistant cancer cells depends on the identification of molecules with drug-like characteristics that can trigger methuosis [14]. Overmeyer et al. [13] discovered indolyl-pyridinyl-propenones (also referred to as indole-based chalcones), which can induce methuosis in glioblastoma cells, including those resistant to temozolomide. They could serve as a model for developing new drugs designed to trigger nonapoptotic cell death in cancers that have become resistant to therapies targeting DNA damage and apoptotic mechanisms.

Our previous study indicated that the exposure of normal brain cells to mephedrone, a synthetic derivative of *S*-cathinone, which is a natural alkaloid present in the leaves of the *Catha edulis* plant, resulted in a modest effect on neuron and astrocyte viability and a significant reduction in oligodendrocyte proliferation [15]. Moreover, it has been shown that mephedrone produces cytotoxicity in SH-SY5Y neuroblastoma cells [16]. To date, no studies have evaluated the effects of mephedrone on glioblastoma cells. In the present study, we investigated the effect of mephedrone on selected human glioblastoma cells (LN-18 and LN-229), human glioblastoma multiforme cells (T98G) and human anaplastic astrocytoma cells (MOGGCCM). The cell lines used for the study were selected considering their genetic and molecular diversity and sensitivity to temozolomide, the main drug used to treat glioblastoma. A detailed summary of the main genetic and molecular features of the investigated cell lines, with particular emphasis on alterations associated with cell growth, proliferation, and survival, is presented in the Supplementary Materials (Table S1 [17,18,19,20,21,22,23,24,25,26,27]).

The effects of mephedrone on cell viability and proliferation, DNA synthesis, cell cycle progression and the type of cell death were studied on these selected cell lines.

## 2. Results

### 2.1. Effect of Mephedrone on the Metabolic Activity of Selected Astrocytoma and Glioblastoma Cells

**Figure 1:**
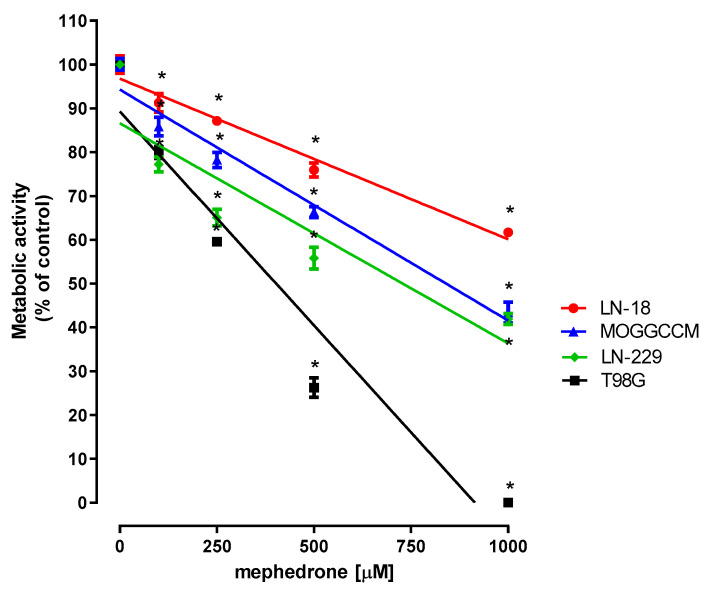
Influence of mephedrone on the metabolic activity of selected human glioblastoma and astrocytoma cell lines. Cells were exposed to culture medium alone (control) or culture medium containing mephedrone (100–1000 µM). Cell metabolic activity was examined after 96 h of cell treatment by means of MTT assay. Data are presented as mean ± SEM of 6 independent trials and were analyzed by means of linear regression, * *p* < 0.05 vs. control; one-way ANOVA test; post hoc test: Dunnett’s.

As presented in Figure 1, the metabolic activity of the human anaplastic astrocytoma cell line MOGGCCM and the glioblastoma cell lines LN-18, LN-229 and T98G in response to mephedrone decreased in a concentration-dependent manner, as measured by means of the MTT assay. The threshold concentrations of mephedrone required to elicit antiproliferative effects in all tested cultures were as low as 100 μM. The highest applied mephedrone concentration (1000 μM) inhibited cell proliferation by 38.34% (LN-18), 56.60% (MOGGCCM), 58.14% (LN-229) and 100% (T98G). The IC_50_ values are presented in Table S2.

### 2.2. Microscopic Detection of Apoptosis, Autophagy, Necrosis and Cytoplasmic Vacuolization

The microscopic observations revealed that mephedrone applied at concentrations ranging from 100 to 1000 µM to the LN-18, LN-229, T98G and MOGGCCM culture media for 24 and 48 h only slightly enhanced the processes of apoptosis in LN-18 and LN-229 while causing virtually no necrosis and autophagy in all the other studied cell lines (Table S3). Notably, in the glioblastoma LN-18 cell line, mephedrone at a concentration of 1000 µM induced strong vacuolization, found in 87% of the cell population after 24 h and 86% after 48 h of incubation (Table 1). In the remaining cell lines, mephedrone induced only trace amounts of vacuolization, with the effect being most pronounced in the case of the T98G cell line.

### 2.3. Effect of Mephedrone on the Proliferation of LN-18 and T98G Cells

**Figure 2:**
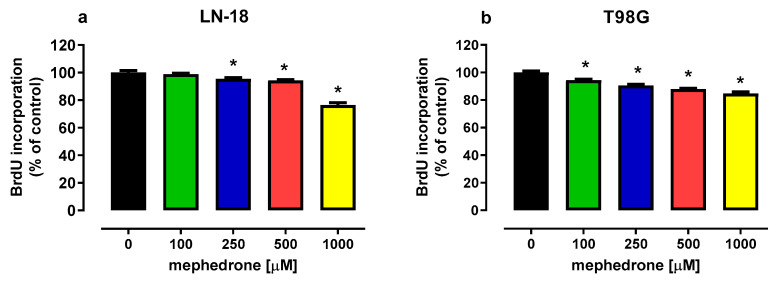
Influence of mephedrone on the proliferation of human glioblastoma LN-18 cells (**a**) and human glioblastoma multiforme T98G cells (**b**). Cells were exposed to culture medium alone (control) or culture medium containing mephedrone at concentrations ranging from 100 to 1000 μM for 48 h. Cell proliferation was measured photometrically by means of the BrdU assay. Results are presented as the mean ± SEM of 6 independent trials, * *p* < 0.05 vs. control; one-way ANOVA test; post hoc test: Dunnett’s.

The results of the BrdU assay, reflecting DNA synthesis, revealed the antiproliferative effect of mephedrone in both human glioblastoma LN-18 cells and human glioblastoma multiforme T98G cells. Mephedrone at concentrations of 250, 500 and 1000 μM decreased BrdU incorporation into newly synthesized DNA in LN-18 cells by 4.68%, 5.78% and 23.65%, respectively (Figure 2a). Mephedrone, in the whole range of investigated concentrations, inhibited the proliferation of T98G cells. In the presence of mephedrone, DNA synthesis in T98G cells declined to the following levels: 94.42% (100 µM), 90.57% (250 µM), 87.97% (500 µM) and 84.75% (1000 µM) (Figure 2b).

### 2.4. Effect of Mephedrone on Cell Cycle Progression in LN-18 and T98G Cells

**Figure 3:**
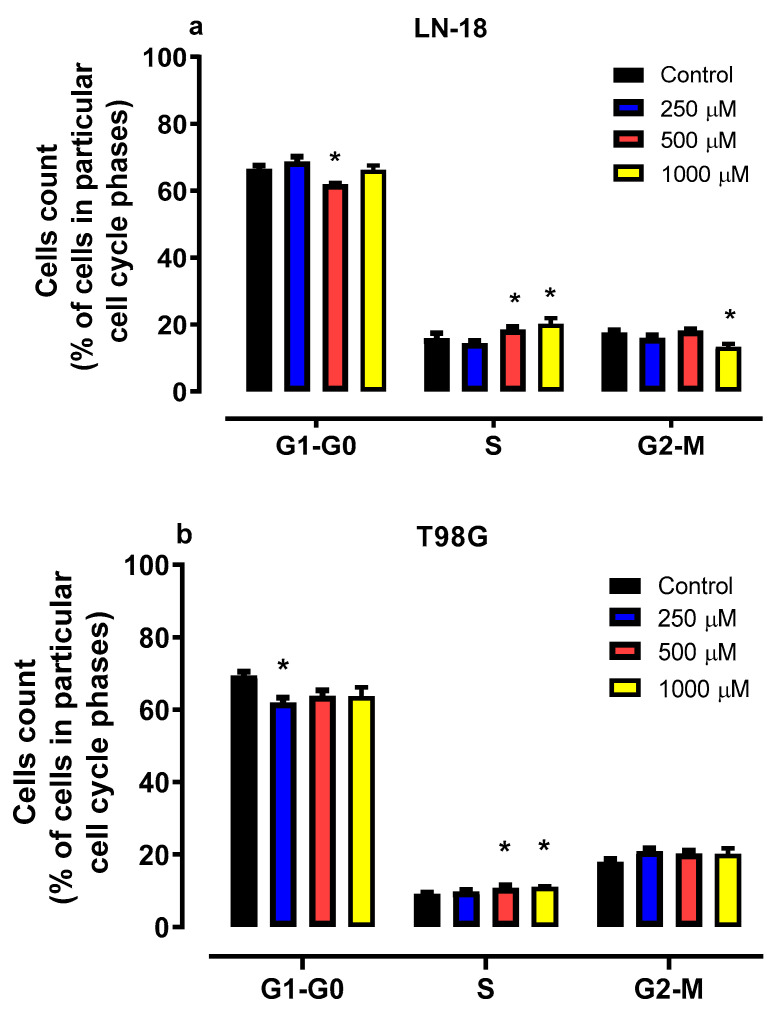
Influence of mephedrone on cell cycle progression in human glioblastoma LN-18 cells (**a**) and human glioblastoma multiforme T98G cells (**b**). Cells were exposed for 48 h to culture medium alone (control) or culture medium containing mephedrone at concentrations of 250, 500 and 1000 μM. DNA content was determined using flow cytometry after cell staining with propidium iodide. Analysis was used to define the percentage of LN-18 cells and T98G cells in G0/G1, S and G2-M cell cycle phases. Data represent the mean ± SEM of 3 independent trials, * *p* < 0.05 vs. control; one-way ANOVA test; post hoc test: Dunnett’s.

As presented in Figure 3a, mephedrone affected cell cycle progression in LN-18 cells. The incubation of cells in the presence of mephedrone at a concentration of 500 µM for 48 h resulted in a decrease in cell number in the G1/G0 phase from 66.50% (control) to 61.93%. At the same time, the number of cells in the S-phase in cells exposed to mephedrone at concentrations 500 and 1000 µM increased from 14.40% in the control conditions to 18.56% and 20.23%, respectively. Moreover, mephedrone at a concentration of 1000 µM reduced the cell number in the G2/M phase from 17.59% (control) to 13.37%.

As presented in Figure 3b, 48 h of T98G cell incubation with mephedrone revealed significant changes in both the G0/G1 and S phases. Mephedrone at a concentration of 250 µM decreased the cell number in the G0/G1 phase to 62.03%, compared to 69.42% in the untreated cells. At the same time, treatment with mephedrone led to a significant S-phase arrest. The number of cells in the S phase increased up to 10.81% (500 µM) and 11.10% (1000 µM) compared to the control (9.15%).

### 2.5. Effect of Mephedrone on LN-18 and T98G Cell Morphology

**Figure 4:**
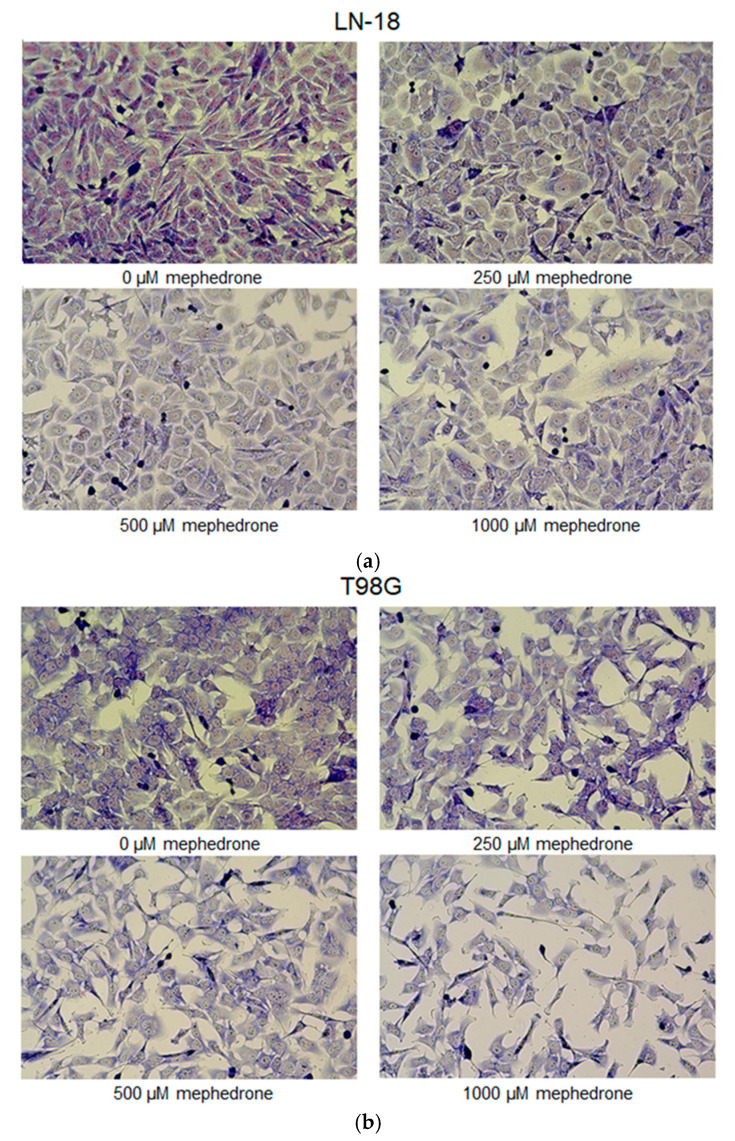
Influence of mephedrone on the morphology of human glioblastoma LN-18 cells (**a**) and human glioblastoma multiforme T98G cells (**b**). Cells were exposed for 48 h to culture medium alone (control) or culture medium containing mephedrone at concentrations of 250, 500 to 1000 μM. Changes in cell morphology were visualized by the May–Grünwald–Giemsa staining method and examined under light microscopy. Magnification: 400×.

Microscopic analysis of LN-18 and T98G cells treated with mephedrone confirmed the antiproliferative properties of the investigated compound, as evidenced by a dose-dependent reduction in the number of human glioblastoma cells (Figure 4). The exposure of cells to mephedrone revealed changes in their intracellular pH homeostasis, since the nuclei of LN-18 cells turned from pink to light blue, while the color of the nuclei in T98G cells changed from light blue to celadon, indicating acidification.

### 2.6. Fluorescence Microscopic and Electron Microscopic Evidence for Vacuolization of LN-18 Cells Exposed to Mephedrone

**Figure 5:**
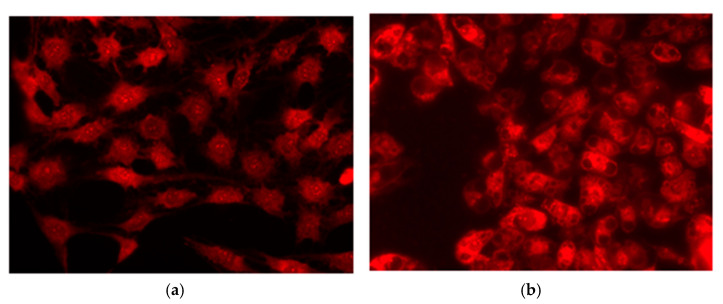
Microscopic evidence for the vacuolization of the human glioblastoma multiforme cell line LN-18 exposed to mephedrone. Untreated cancer cells (control) (**a**), as well as cancer cells (**b**), exposed to mephedrone at a concentration of 1000 µM for 48 h were stained with acridine orange and examined under fluorescence microscopy. Representative pictures are presented. Magnification: 400×.

**Figure 6:**
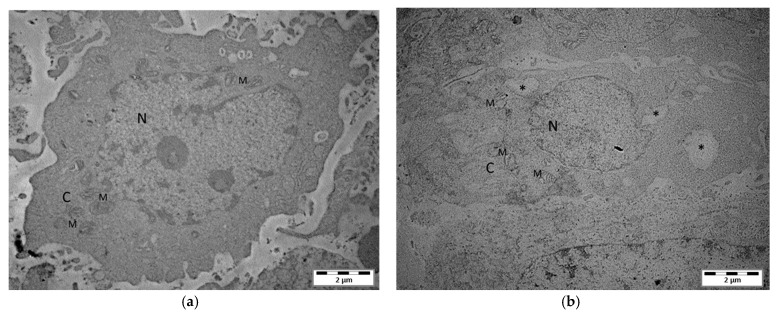
Electron microscopic evidence for the vacuolization of the human glioblastoma multiforme cell line LN-18 exposed to mephedrone. Untreated control cells (**a**) and LN-18 cells after 24 h incubation with mephedrone (1000 µM) (**b**). N—nucleus; C—cytoplasm; M—mitochondria. * Enlarged vacuoles. Magnification: 8000×.

Fluorescence microscopic detection revealed that mephedrone at a concentration of 1000 µM induced strong vacuolization of the cytoplasm compared to the control cells (Figure 5). Electron microscopy studies confirmed these observations, showing large electron lucent cytoplasmic vacuoles in the treated LN-18 cells but not in the controls. In addition, morphological changes in mitochondria characterized by a reduction in electron density in the matrix can be observed after mephedrone treatment (Figure 6).

### 2.7. The Cytotoxic Effect of Mephedrone in LN-18 and T98G Cells

As presented in Figure S1, mephedrone was cytotoxic for human glioblastoma LN-18 and T98G cells, the sign of which was an increased release of LDH into the culture medium through damaged cancer cell membranes. The most significant changes were observed in LN-18 cells (Figure S1a), wherein the LDH level increased from 105.6% (100 µM mephedrone) to 111.8% (1000 µM mephedrone). In the case of the T98G cell line (Figure S1b), only the highest concentration of mephedrone (1000 µM) induced a statistically significant cytotoxic effect, causing the release of LDH up to a level of 126.6%.

## 3. Discussion

Our results revealed that mephedrone possesses potential anticancer activity. The viability and proliferation of all four selected human glioblastoma and human anaplastic astrocytoma cell lines were decreased in a concentration-dependent manner. The T98G line appeared to be the most sensitive to mephedrone, followed by LN-229 and MOGGCCM, and the least sensitive was LN-18. A microscopic evaluation of the nature of cell death showed that mephedrone only slightly enhances the processes of apoptosis in LN-18 and LN-229, while it causes virtually no necrosis and autophagy in all other studied cell lines. Importantly, it was revealed that mephedrone induced substantial cytoplasmic vacuolization of the human glioblastoma LN-18 cell line and less pronounced vacuolization of the human glioblastoma multiforme T98G cell line. Thus, further in-depth studies were performed on these cell lines. Additional studies conducted on LN-18 and T98G cells confirmed the significant antiproliferative properties of mephedrone, which reduced DNA synthesis and affected cell cycle progression, implying significant S-phase arrest. Furthermore, May–Grünwald–Giemsa staining of the LN-18 and T98G cells treated with mephedrone visualized the antiproliferative effect of the tested compound.

Similarly, in our previous study, we showed that mephedrone reduced the viability of neurons derived from the human neuroblastoma cell line SH-SY5Y and rat astrocytes while not affecting rat oligodendrocytes in the cell line OLN-93. Nevertheless, the decrease in neuron and astrocyte viability in response to mephedrone, even at the highest tested concentration (1000 µM), was discreet. On the contrary, mephedrone revealed a significant antiproliferative effect on both astrocytes and oligodendrocytes [15]. However, the previously described inhibition of astrocyte proliferation was significantly less than the changes currently reported in both human glioblastoma and astrocytoma cell lines. This was evidenced by the significantly lower IC_50_ values determined from the results of the MTT assay carried out in normal and cancer cells after 96 h of exposure to mephedrone in comparable concentration ranges. In contrast, the above comparison of IC_50_ values revealed that OLN-93 cells are as sensitive to mephedrone as the glioblastoma cell line LN-229 but significantly more resistant than the glioblastoma multiforme cell line T98G. It has also been shown that mephedrone produced cytotoxicity in the SH-SY5Y neuroblastoma cell line and reduced mitochondrial respiration [16], while, as previously indicated, the metabolic activity of neurons derived from SH-SY5Y cells was only slightly affected by mephedrone [15]. This may suggest that the differentiation of cancer cells into normal cells increases their resistance to the antiproliferative effects of mephedrone.

In a recent study, Alanazi et al. [28] investigated the effect of mephedrone on human neuroblastoma (SH-SY5Y) and astrocytoma (U-373) cells and found that mephedrone stimulated metabolic processes in U-373 cells but not in SH-SY5Y cells at concentrations of up to 100 µM. However, at higher concentrations, this effect was reversed, reducing the proliferation of the cells tested, an effect similar to the results of our study. Differently to our study, they found no changes in the morphological structure of the cells. It is difficult to indicate the reason for the differences between the results of Alanazi et al. [28] and our findings. This may be due to differences in study design, but a detailed comparison is not possible due to insufficient description of the methods used. The difference may also be due to the different sensitivities of various types of cancer cells to the effects of mephedrone. It should be noted that in our study, the differences in IC_50_ for individual cells varied widely from 277 to 1728 µM. Moreover, unusually large vacuolization occurred only in LN-18 cells, whereas it was negligible in the other cells tested. These findings indicate that the effect of mephedrone on glioblastoma and astrocytoma is cell-specific.

Interestingly, in vivo studies indicated the inhibition of cell proliferation and an increase in apoptosis in the hippocampus of pups delivered by mice treated subcutaneously with mephedrone on a repeated schedule during pregnancy [29]. Data on the antiproliferative activity of mephedrone and its mechanism of action are sparse. In a study by Soares et al. [30] conducted on SH-SY5Y neuroblastoma cells, mephedrone-induced oxidative stress was assessed with increasing intracellular levels of reactive oxygen species (ROS) and decreasing intracellular glutathione levels. Pre-treatment with trolox (antioxidant) and *N*-acetyl-l-cysteine (antioxidant and glutathione precursor) partially or completely inhibited these effects of mephedrone. Moreover, mephedrone caused mitochondrial dysfunction through the depolarization of the mitochondrial membrane and intracellular depletion of adenosine triphosphate (ATP). Apoptosis and autophagy were also activated by mephedrone, but pre-incubation with bafilomycin A1, an inhibitor of the vacuolar H^+^-ATPase, did not protect against the cytotoxicity induced by mephedrone [30].

Although our studies revealed the weak proapoptotic abilities of mephedrone in the investigated cell lines, autophagy and necrosis were not detected. The resistance discovered to both apoptosis and autophagy induction could be associated with missense mutations in *TP53*, presented in all investigated cell lines [19,20]. The cell lines most resistant to the indicated cell death types were MOGGCCM and T98G cells, which contain mutations in DNA-binding domains (A159V and M237I, respectively), leading to decreased p53 transcriptional activity and increased resistance to apoptosis [31,32]. On the contrary, the LN-229 cell line, which was the most sensitive to apoptosis and autophagy induction, contained a mutation at the very end of the proline-rich domain (P98L); however, the molecular consequences of this remain unknown. Mutations in *TP53* (C238S, M237I), as well as the deletion of *CDKN2A* present in LN-18 and T98G [19,20], could also explain the failure to induce cell cycle arrest in the G0-G1 phase and cell accumulation in the S-phase in response to mephedrone [33,34]. It is also worth mentioning two other missense mutations that strongly influence signal transduction in the Akt pathway associated with the promotion of cell growth and survival. In the cell line LN-18, a mutation in *PIK3CB* (*E1051K*) was reported, which accelerated the coding of phosphatidylinositol 3-kinase, and this may explain the increased resistance of these cells to the cytotoxic effects of mephedrone, as well as attempts to reduce metabolic activity or cell death [19,20,35,36]. On the contrary, the silencing mutation in *PTEN* (*L42R*) occurring in the cell line T98G, which codes lipid phosphatase, which antagonizes the action of PI3K, may explain the strongest sensitivity of the indicated cells to both the cytotoxic and antiproliferative effects of mephedrone [19,20,36,37].

When discussing the differences in the response of the tested glioblastoma cell lines to mephedrone, it is necessary to take into account changes in the methylation level of the *MGMT* promoter, which is a useful tool for predicting the treatment outcome for patients with glioblastoma undergoing chemotherapy with alkylating drugs, including temozolomide [38]. Indeed, the indicated feature determined the selection of glioblastoma cell lines for the presented studies. As presented by Schnöller et al. [39], the highest *MGMT* promoter of methylation was observed in LN-229 cells, and the lowest was observed in LN-18 cells, while T98G cells represented an intermediate state. Since this methylation reaction is the key mechanism of *MGMT* gene silencing, the lowest expression of the DNA repair enzyme O6-methylguanine-DNA methyltransferase was recorded in LN-229, while it was weak in T98G and high in LN-18 cells. Furthermore, a positive correlation between resistance to temozolomide treatment and the methylation level of *MGMT* was revealed [39]. Thus, our results provide an additional argument for using MGMT expression as a biomarker for predicting tumor sensitivity to treatment.

Our microscopic studies performed on human glioblastoma LN-18 cells exposed to mephedrone for 24 and 48 h discovered strong vacuolization, observed in 87% and 86% of the cell population, respectively. Electron microscopy studies disclosed large electron lucent cytoplasmic vacuoles in the treated LN-18 cells, confirming this phenomenon. Much less pronounced vacuolization was also revealed in T98G cells exposed to mephedrone. Cytoplasmic vacuolization is a well-known morphological phenomenon observed in mammalian cells that develops spontaneously or after exposure to bacterial or viral infections, in addition to various natural and synthetic low-molecular-weight substances [40]. The cytoplasmic vacuolization of mammalian cells can either be transient or permanent. The transient form occurs in non-cytotoxic conditions and has a reversible impact on cell cycle progression and migration [41]. Transient vacuolization is typically not linked to cell death. In contrast, irreversible vacuolization denotes cytopathological conditions that result in cell death, provided that the cytotoxic stimulus continues. Vacuoles are generated in various cellular compartments through different mechanisms, and the ability to trigger irreversible cytoplasmic vacuolization has been shown for a range of natural and synthetic substances, including pharmaceuticals and industrial pollutants [42]. Some inducers of irreversible vacuolization cause nonapoptotic cell death, including methuosis, paraptosis, oncosis and necroptosis [43,44]. Importantly, these types of cell death are typical of cancer cells, including cells that are resistant to apoptosis, which makes research promising for the development of new therapeutic strategies for cancer treatment [42].

The accumulated data seem to indicate that in the glioblastoma LN-18 cell line, mephedrone causes substantial cytoplasmic vacuolization, which can lead to methuosis. On the other hand, vacuolization may be the result of the activation of macropinocytosis, which is a cell defense mechanism triggered under conditions of insufficient nutrition [45]. During this endocytic pathway, cells can take up extracellular fluid, forming macropinosomes that fuse with late endosomes. As a consequence, methuosis may occur to block this fusion by inducing the formation of large vacuoles, which leads to the rupture of the plasma membrane [46].

To obtain an insight into whether this mephedrone-induced phenomenon in the LN-18 cell line leads to methuosis, we performed an LDH assay, assuming that if vacuolization leads to cell disintegration, then the LDH levels should rise [47]. Although we noted an increased level of LDH released by damaged cancer cell membranes, its magnitude and dynamics did not equate to the severity of vacuolization (Figure S1a). In addition, it should be emphasized that cells of the LN-18 line with substantial vacuolization are the least sensitive to mephedrone in the MTT assay, which may indicate that, in this case, vacuolization is a defense mechanism. If mephedrone-induced cytoplasmic vacuolization does indeed occur, or it is combined with another agent/therapy, this could result in methuosis, which would open up new avenues for the use of mephedrone or its derivatives in the treatment of gliomas.

Considering the response of the studied glioma cell lines to mephedrone, the role of metabolic features and the tumor microenvironment should also be taken into account. As shown by Alanazi et al., mephedrone affects cell membrane function due to the depletion of ether lipids [28]. Therefore, knowing the molecular profile of cell lines that exhibit similar biological behavior to primary tumors may prove necessary to define a new therapeutic approach. Mephedrone can also serve as a chemical structure, providing a starting point for the synthesis of new compounds with antiproliferative activity, but without many of the side effects of the parent drug.

It should be noted that mephedrone easily crosses the blood–brain barrier, which is an essential precondition for treating cancer located in the brain. Additionally, the use of some of the most commonly used drugs in cancer therapy, doxorubicin and methotrexate, is limited in part due to their inability to cross the blood–brain barrier. New synthetized mephedrone derivatives could increase blood–brain barrier permeability and could be used as a pharmacological tool to enable potential therapeutic drugs to reach the brain.

Mephedrone is a cathinone that produces stimulant and empathogenic effects similar to amphetamines, methylamphetamine, cocaine and 3,4-methylenedioxymethamphetamine (MDMA) [48]. It interacts with monoamine plasma membrane transporters for serotonin, dopamine, and likely also norepinephrine, stimulating their release and blocking their reuptake [49,50]. The drug enhances the feeling of excitement, euphoria and empathy, promotes talkativeness, and increases libido, concentration and alertness while also inducing a general sense of heightened arousal [51]. The subjective effects last around one hour due to the short half-life and rapid metabolism of mephedrone [52]. Side effects reported by mephedrone users include, in order of occurrence, bruxism (grinding the teeth and clenching the jaw), paranoia, sore nasal passages and nose bleed after insufflation, hot flushes, memory loss, insomnia, depression, vomiting, chest pains and skin rashes [53]. Human studies highlight that cognitive function is worsened in mephedrone users, involving deterioration in verbal learning, verbal fluency and cognition flexibility compared to non-users [54]. Although animal studies have confirmed that mephedrone causes memory impairment, its mechanism is not fully understood [55,56]. One hypothesis assumes that mephedrone induces memory impairment due to its neurotoxic effects, but this remains contradictory [57]. In our previous study, binge-like mephedrone treatment induced delayed long-lasting memory impairments assessed in the passive avoidance test in mice [15].

When considering all the above objections to the use of mephedrone in the treatment of glioblastoma, its use seems to be controversial due to its addictive potency and potential considerable side effects. Regardless, in the absence of satisfactory effective therapy for this cancer, such a treatment does appear to be justified. In medicine, there are examples of substances commonly recognized as addictive which are used in pharmacotherapy. Opioids are drugs prescribed to treat not only moderate and severe postoperative pain and cancer pain but also to treat cough, shortness of breath and diarrhea [58]. Amphetamines are used to treat obesity, narcolepsy or attention-deficit hyperactivity disorder (ADHD) [59,60]. Cannabinoid-containing drugs may be beneficial in treating specific rare forms of epilepsy, nausea and vomiting related to cancer chemotherapy, as well as appetite and weight loss associated with HIV/AIDS [61]. Attempts have also been made to use the above-mentioned drugs in anticancer therapy.

More recently, the treatment of resistant glioblastoma stem cells with methadone, a μ-opioid receptor agonist, in combination with doxorubicin, has been shown to strongly induce apoptosis, proving the ability of this drug to sensitize glioma stem cells to doxorubicin and overcome the chemotherapy resistance of glioblastoma stem cells. Moreover, it has been revealed that the activation of opioid receptors by methadone significantly inhibited tumor growth in an in vivo nude mouse model [62]. In addition, specific cannabinoids have also been investigated in numerous preclinical glioma models [63]. Interestingly, a Phase II study has been approved to determine whether adding a cannabinoid (nabiximol) to standard temozolomide therapy improves glioblastoma treatment.

In conclusion, our study indicated that mephedrone possesses potential anticancer activity against selected human glioblastoma and human anaplastic astrocytoma cell lines. The anticancer effect of mephedrone was mainly connected with impaired cellular metabolism, DNA synthesis and cell cycle progression, as observed in the glioblastoma LN-18 cell line and the glioblastoma multiforme T98G cell line. Furthermore, we observed substantial vacuolization in the LN-18 cell line in response to mephedrone treatment, which suggests that the induction of methuosis, a nonapoptotic type of cell death, could be promising for the development of new therapeutic approaches to cancer. The relatively high IC_50_ values and high effective concentrations of mephedrone obtained in this study do not rule out a beneficial effect of mephedrone in vivo. Findings from similar studies using temozolomide on glioma cell lines suggest that results obtained in vitro do not necessarily translate to effective treatment outcomes in vivo (see comparison of IC_50_ values in the Supplementary materials, Table S4 [64,65,66]).

Thus, further in-depth research is needed to confirm the possible use of mephedrone or its derivatives in glioma therapy. Further studies should consider that various types of neuroblastoma and astrocytoma cells respond differently to mephedrone, and therefore its effects should not be generalized.

## 4. Materials and Method

### 4.1. Reagents

Mephedrone ((RS)-2-methylamino-1-(4-methylphenyl) propan-1-one was purchased from Tocris Bioscience, Bristol, UK. Other reagents for in vitro studies were of the highest commercially available purity, purchased from Sigma-Aldrich, St. Louis, MO, USA.

### 4.2. Cell Lines

Human glioblastoma multiforme cells (T98G, ECACC 92090213) and human anaplastic astrocytoma cells (MOGGCCM, ECACC 86022702) were grown in a 3:1 mixture of Dulbecco’s Modified Eagle Medium (DMEM) and Ham’s nutrient mixture F-12 (Sigma-Aldrich, St. Louis, MO, USA) supplemented with 10% fetal bovine serum (FBS). LN-18 and LN-229 (human glioblastoma, ATCC, CRL-2610 and CRL-2611) were cultured in DMEM with 5% FBS. All cell lines were supplemented with 100 IU/mL penicillin and 100 µg/mL streptomycin. The cultures were kept at 37 °C in a humidified atmosphere of 95% air and 5% CO2. The main genetic and molecular features of the above-mentioned cell lines are presented in the Supplementary Materials (Table S1).

### 4.3. MTT Assay—Assessment of Cell Metabolic Activity

Cells were seeded on 96-well microplates at a density of 2 × 10^4^ cells/mL. On the following day, the culture medium was removed, and cells were exposed to mephedrone at concentrations ranging from 100 to 1000 µM. Tested solutions were prepared in medium supplemented with 10% FBS. Cell metabolic activity was assessed after 96 h of incubation by means of an MTT assay, as previously described [15]. Based on the MTT results, the IC_50_ value (concentration causing 50% proliferation inhibition compared to the control) was calculated according to the Litchfield and Wilcoxon method [67] using GraphPad Prism 5.

### 4.4. Microscopic Detection of Apoptosis, Autophagy, Necrosis and Cytoplasmic Vacuolization with Fluorochromes

Cells were incubated with mephedrone at concentrations ranging from 100 to 1000 µM for 24 h and 48 h. For the identification of apoptosis and necrosis in the MOGGCCM, T98G, LN-229, and LN-18 cell lines, a staining method with Hoechst 33,342 (Sigma-Aldrich, St. Louis, MO, USA) and propidium iodide (Sigma) was chosen, as described previously [68]. In the case of autophagy, staining with acridine orange (AO) to detect typical acidic vesicular organelles (AVOs) was performed. The same method facilitated the observation of the enlarged vacuoles within the cells [68]. To analyze cell death morphology, we employed a methodology outlined in earlier studies [69]. Each experiment was conducted independently in triplicate.

### 4.5. BrdU Assay—Cell Proliferation Assessment

LN-18 and T98G cells were seeded on 96-well microplates at a density of 5 × 10^4^ cells/mL (100 µL/well). The next day, the culture medium was removed and cells were exposed to mephedrone at concentrations of 100, 250, 500 and 1000 μM. Untreated cells were used as the control. Cell proliferation was quantified after 48 h of treatment using Cell Proliferation ELISA BrdU (Roche Diagnostics GmbH, Penzberg, Germany), according to the manufacturer’s instruction. Absorbance was recorded on a microplate reader (BioTek ELx800, Highland Park, Winooski, Vermont, VT, USA) at a 450 nm wavelength. The influence of mephedrone on DNA synthesis was presented as the percentage of BrdU incorporation versus the control cells (indicated as 100%).

### 4.6. Flow Cytometry—Evaluation of Cell Cycle Progression

LN-18 and T98G cells were seeded on 6-well microplates at a density of 5 × 10^4^ cells/mL (1 mL/well). On the following day, the culture medium was removed, and the cells were exposed for 48 h to the mephedrone at concentrations of 250, 500 and 1000 µM. Untreated cells were used as a control. The cells were analysed on the FacsCanto instrument (Becton Dickinson, San Jose, CA, USA). A comprehensive explanation of the methodology can be found in a prior publication [68].

### 4.7. May–Grünwald–Giemsa Staining—Examination of Cell Morphology

LN-18 and T98G cells were seeded on Lab-Tek Chambers Slides at a density of 5 × 10^4^ cells/mL (450 μL/well). The next day, the culture medium was removed, and the cells were exposed to mephedrone at concentrations of 250, 500 and 1000 μM. Untreated cells were used as a control. After 48 h of treatment with the investigated compound, the cells were stained by the May–Grünwald–Giemsa method. Alterations in cell morphology were examined using inverted light microscope OPTA-TECH MW50 (OPTA-TECH, Warsaw, Poland).

### 4.8. Transmission Electron Microscopy

The samples were observed under a Zeiss Libra 120 transmission electron microscope (Carl Zeiss SMT AG, Oberkochen, Germany). Electron microscopic analysis was performed according to the method described in our previous study [69].

### 4.9. LDH Assay—Cytotoxicity Assessment

A cytotoxicity detection kit based on the measurement of lactate dehydrogenase (LDH) activity was applied (In Vitro Toxicology Assay Kit, Lactate Dehydrogenase-Based, Sigma-Aldrich, St. Louis, MO, USA). Cells were seeded on 96-well microplates at a density of 5 × 10^4^ cells/mL. On the following day, the culture medium was replaced by a fresh one, alone or with mephedrone at concentrations ranging from 100 to 1000 µM. The tested solutions were prepared in medium supplemented with 2% FBS. The LDH assay was conducted following the procedure outlined in our earlier study [70]. The results were presented as a percentage of LDH released from cells treated with the tested mephedrone concentrations versus cells grown in the control medium (indicated as 100%).

### 4.10. Statistical Analysis

Data were presented as the mean value and standard error of the mean (SEM). Statistical analysis was performed using one way-ANOVA with the Dunnett’s post hoc test. The LDH assay was presented using estimation graphics according to Ho et al. [71]. Significance was accepted at *p* < 0.05.

## Figures and Tables

**Table 1:** Percentage of cells displaying vacuolization after 24 and 48 h of incubation in the presence of mephedrone. Data are presented as the percentage of cells in which vacuolization was observed. Mephedrone 0 µM means control.

Cell Line	Mephedrone [µM]									
	0	100	250	500	1000	0	100	250	500	1000
	Incubation Time 24 h					Incubation Time 48 h				
LN-18	0	3	14	72	87	0.5	12	24	81	86
LN-229	0	0	0	0	2.7	0	0	0	0.2	0.9
T98G	0	0	0.5	2.1	3.7	0	0	0.7	2.7	2.9
MOGGCCM	0	0	0.5	1.9	2.7	0.1	0	0.9	1.2	2.1

## Data Availability

The data presented in this study are available from the corresponding author.

## Supplementary Materials

The following supporting information can be downloaded at https://www.mdpi.com/article/10.3390/molecules30112277/s1. Figure S1. Cytotoxic effect of mephedrone in human glioblastoma LN-18 cells (a) and human glioblastoma multiforme T98G cells (b). Table S1. Summary of the main genetic and molecular features of investigated cell lines with particular emphasis on alterations associated with cell growth, proliferation and survival. Table S2. IC_50_ values of mephedrone were calculated for selected human glioblastoma and astrocytoma cell lines as well as rat oligodendrocytes based on results of MTT assays performed after 96 h of cell treatment. Table S3. Percentage of cells displaying apoptosis, necrosis and autophagy after 24 and 48 h of incubation in the presence of mephedrone. Table S4. The concentration (μM) of temozolomide and mephedrone in gliomas cell lines.

## References

[1] Peng F., Liao M., Qin R., Zhu S., Peng C., Fu L., Chen Y., Han B. (2022). Regulated cell death (RCD) in cancer: Key pathways and targeted therapies. Signal Transduct. Target. Ther..

[2] Dumontet C., Jordan M.A. (2010). Microtubule-binding agents: A dynamic field of cancer therapeutics. Nat. Rev. Drug Discov..

[3] Schonthal A.H. (2013). Pharmacological targeting of endoplasmic reticulum stress signaling in cancer. Biochem. Pharmacol..

[4] Abdullah L.N., Chow E.K. (2013). Mechanisms of chemoresistance in cancer stem cells. Clin. Transl. Med..

[5] Thakkar J.P., Dolecek T.A., Horbinski C., Ostrom Q.T., Lightner D.D., Barnholtz-Sloan J.S., Villano J.L. (2014). Epidemiologic and molecular prognostic review of glioblastoma. Cancer Epidemiol. Biomark. Prev..

[6] Ma Y., Wang Y., Nie C., Lin Y. (2023). The efficacy of targeted therapy combined with radiotherapy and temozolomide-based chemotherapy in the treatment of glioma: A systemic review and meta-analysis of phase II/III randomized controlled trials. Front. Oncol..

[7] Ortiz R., Perazzoli G., Cabeza L., Jiménez-Luna C., Luque R., Prados J., Melguizo C. (2021). Temozolomide: An updated overview of resistance mechanisms, nanotechnology advances and clinical applications. Curr. Neuropharmacol..

[8] Silantyev A.S., Falzone L., Libra M., Gurina O.I., Kardashova K.S., Nikolouzakis T.K., Nosyrev A.E., Sutton C.W., Mitsias P.D., Tsatsakis A. (2019). Current and future trends on diagnosis and prognosis of glioblastoma: From molecular biology to proteomics. Cells.

[9] Omuro A., DeAngelis L.M. (2013). Glioblastoma and other malignant gliomas: A clinical review. JAMA.

[10] Wang X., Hua P., He C., Chen M. (2022). Non-apoptotic cell death-based cancer therapy: Molecular mechanism, pharmacological modulators, and nanomedicine. Acta Pharm. Sin. B.

[11] Button R.W., Roberts S.L., Willis T.L., Hanemann C.O., Luo S. (2017). Accumulation of autophagosomes confers cytotoxicity. J. Biol. Chem..

[12] Overmeyer J.H., Kaul A., Johnson E.E., Maltese W.A. (2008). Active ras triggers death in glioblastoma cells through hyperstimulation of macropinocytosis. Mol. Cancer Res..

[13] Overmeyer J.H., Young A.M., Bhanot H., Maltese W.A. (2011). A chalcone-related small molecule that induces methuosis, a novel form of non-apoptotic cell death, in glioblastoma cells. Mol. Cancer.

[14] Robinson M.W., Overmeyer J.H., Young A.M., Erhardt P.W., Maltese W.A. (2012). Synthesis and evaluation of indole-based chalcones as inducers of methuosis, a novel type of nonapoptotic cell death. J. Med. Chem..

[15] Marszalek-Grabska M., Zakrocka I., Budzynska B., Marciniak S., Kaszubska K., Lemieszek M.K., Winiarczyk S., Kotlinska J.H., Rzeski W., Turski W.A. (2022). Binge-like mephedrone treatment induces memory impairment concomitant with brain kynurenic acid reduction in mice. Toxicol. Appl. Pharmacol..

[16] den Hollander B., Sundström M., Pelander A., Ojanperä I., Mervaala E., Korpi E.R., Kankuri E. (2014). Keto amphetamine toxicity—Focus on the redox reactivity of the cathinone designer drug mephedrone. Toxicol. Sci..

[17] https://www.atcc.org/.

[18] https://www.culturecollections.org.uk/.

[19] https://www.cellosaurus.org/index.html.

[20] https://ckb.jax.org/.

[21] Jakubowicz-Gil J., Bądziul D., Langner E., Wertel I., Zając A., Rzeski W. (2017). Temozolomide and sorafenib as programmed cell death inducers of human glioma cells. Pharmacol. Rep..

[22] Sumorek-Wiadro J., Zając A., Bądziul D., Langner E., Skalicka-Woźniak K., Maciejczyk A., Wertel I., Rzeski W., Jakubowicz-Gil J. (2020). Coumarins modulate the anti-glioma properties of temozolomide. Eur. J. Pharmacol..

[23] Lee S.Y., Liu S., Mitchell R.M., Slagle-Webb B., Hong Y.S., Sheehan J.M., Connor J.R. (2011). HFE polymorphisms influence the response to chemotherapeutic agents via induction of p16INK4A. Int. J. Cancer.

[24] Hermisson M., Klumpp A., Wick W., Wischhusen J., Nagel G., Roos W., Kaina B., Weller M. (2006). O^6^-methylguanine DNA methyltransferase and p53 status predict temozolomide sensitivity in human malignant glioma cells. J. Neurochem..

[25] St-Coeur P.D., Poitras J.J., Cuperlovic-Culf M., Touaibia M., Morin P. (2015). Investigating a signature of temozolomide resistance in GBM cell lines using metabolomics. J. Neuro-Oncol..

[26] Schnöller L.E., Albrecht V., Brix N., Nieto A.E., Fleischmann D.F., Niyazi M., Hess J., Belka C., Unger K., Lauber K. (2022). Integrative analysis of therapy resistance and transcriptomic profiling data in glioblastoma cells identifies sensitization vulnerabilities for combined modality radiochemotherapy. Radiat. Oncol..

[27] Avellaneda Matteo D., Grunseth A.J., Gonzalez E.R., Anselmo S.L., Kennedy M.A., Moman P., Scott D.A., Hoang A., Sohl C.D. (2017). Molecular mechanisms of isocitrate dehydrogenase 1 (IDH1) mutations identified in tumors: The role of size and hydrophobicity at residue 132 on catalytic efficiency. J. Biol. Chem..

[28] Alanazi I.M., Alzahrani A.R., Alsaad M.A., Moqeem A.L., Hamdi A.M., Taher M.M., Watson D.G., Grant M.H. (2024). The effect of mephedrone on human neuroblastoma and astrocytoma cells. Saudi Pharm. J..

[29] Naseri G., Fazel A., Golalipour M.J., Haghir H., Sadeghian H., Mojarrad M., Hosseini M., Sabzevar S.S., Beheshti F., Ghorbani A. (2018). Exposure to mephedrone during gestation increases the risk of stillbirth and induces hippocampal neurotoxicity in mice offspring. Neurotoxicol. Teratol..

[30] Soares J., Costa V.M., Gaspar H., Santos S., Bastos M.L., Carvalho F., Capela J.P. (2020). Adverse outcome pathways induced by 3,4-dimethylmethcathinone and 4-methylmethcathinone in differentiated human SH-SY5Y neuronal cells. Arch. Toxicol..

[31] Slovackova J., Smarda J., Smardova J. (2012). Roscovitine-induced apoptosis of H1299 cells depends on functional status of p53. Neoplasma.

[32] Menendez D., Inga A., Resnick M.A. (2010). Estrogen receptor acting in cis enhances WT and mutant p53 transactivation at canonical and noncanonical p53 target sequences. Proc. Natl. Acad. Sci. USA.

[33] Boettcher S., Miller P.G., Sharma R., McConkey M., Leventhal M., Krivtsov A.V., Giacomelli A.O., Wong W., Kim J., Chao S. (2019). A dominant-negative effect drives selection of TP53 missense mutations in myeloid malignancies. Science.

[34] Park J.W., Kang J., Lim K.Y., Kim H., Kim S.I., Won J.K., Park C.-K., Park S.-H. (2021). The prognostic significance of p16 expression pattern in diffuse gliomas. J. Pathol. Transl. Med..

[35] Whale A.D., Colman L., Lensun L., Rogers H.L., Shuttleworth S.J. (2017). Functional characterization of a novel somatic oncogenic mutation of PIK3CB. Signal Transduct. Target. Ther..

[36] Hoxhaj G., Manning B.D. (2020). The PI3K-AKT network at the interface of oncogenic signalling and cancer metabolism. Nat. Rev. Cancer.

[37] Hill V.K., Kim J.S., James C.D., Waldman T. (2017). Correction of PTEN mutations in glioblastoma cell lines via AAV-mediated gene editing. PLoS ONE.

[38] Della Monica R., Cuomo M., Buonaiuto M., Costabile D., Franca R.A., Del Basso De Caro M., Catapano G., Chiariotti L., Visconti R. (2022). MGMT and whole-genome DNA methylation impacts on diagnosis, prognosis and therapy of glioblastoma multiforme. Int. J. Mol. Sci..

[39] Schnöller L.E., Piehlmaier D., Weber P., Brix N., Fleischmann D.F., Nieto A.E., Selmansberger M., Heider T., Hess J., Niyazi M. (2023). Systematic in vitro analysis of therapy resistance in glioblastoma cell lines by integration of clonogenic survival data with multi-level molecular data. Radiat. Oncol..

[40] Aki T., Nara A., Uemura K. (2012). Cytoplasmic vacuolization during exposure to drugs and other substances. Cell Biol. Toxicol..

[41] Morissette G., Lodge R., Marceau F. (2008). Intense pseudotransport of a cationic drug mediated by vacuolar ATPase: Procainamide-induced autophagic cell vacuolization. Toxicol. Appl. Pharmacol..

[42] Shubin A.V., Demidyuk I.V., Komissarov A.A., Rafieva L.M., Kostrov S.V. (2016). Cytoplasmic vacuolization in cell death and survival. Oncotarget.

[43] Sperandio S., Poksay K., de Belle I., Lafuente M.J., Liu B., Nasir J., Bredesen D.E. (2004). Paraptosis: Mediation by MAP kinases and inhibition by AIP-1/Alix. Cell Death Differ..

[44] Christofferson D.E., Yuan J. (2010). Necroptosis as an alternative form of programmed cell death. Curr. Opin. Cell Biol..

[45] Song S., Zhang Y., Ding T., Ji N., Zhao H. (2021). The dual role of macropinocytosis in cancers: Promoting growth and inducing methuosis to participate in anticancer therapies as targets. Front. Oncol..

[46] Dekker N.J., Laraia L. (2023). Making bubbles: Targeting VPS41 induces vacuolization and methuosis. Cell Chem. Biol..

[47] Gao X., Ruan X., Ji H., Peng L., Qiu Y., Yang D., Song X., Ji C., Guo D., Jiang S. (2020). Maduramicin triggers methuosis-like cell death in primary chicken myocardial cells. Toxicol. Lett..

[48] Schifano F., Albanese A., Fergus S., Stair J.L., Deluca P., Corazza O., Davey Z., Corkery J., Siemann H., Scherbaum N. (2011). Mephedrone (4-methylmethcathinone; ‘meow meow’): Chemical, pharmacological and clinical issues. Psychopharmacology.

[49] Baumann M.H., Ayestas M.A., Partilla J.S., Sink J.R., Shulgin A.T., Daley P.F., Brandt S.D., Rothman R.B., E Ruoho A., Cozzi N.V. (2012). The designer methcathinone analogs, mephedrone and methylone, are substrates for monoamine transporters in brain tissue. Neuropsychopharmacology.

[50] Kolanos R., Sakloth F., Jain A.D., Partilla J.S., Baumann M.H., Glennon R.A. (2015). Structural modification of the designer stimulant α-pyrrolidinovalerophenone (α-PVP) influences potency at dopamine transporters. ACS Chem. Neurosci..

[51] Winstock A., Mitcheson L., Ramsey J., Davies S., Puchnarewicz M., Marsden J. (2011). Mephedrone: Use, subjective effects and health risks. Addiction.

[52] Green A.R., King M.V., Shortall S.E., Fone K.F.C. (2014). The preclinical pharmacology of mephedrone; not just MDMA by another name. Br. J. Pharmacol..

[53] James D., Adams R.D., Spears R., Cooper G., Lupton D.J., Thompson J.P., Thomas S.H.L. (2011). Clinical characteristics of mephedrone toxicity reported to the UK National Prisons Information Service. Emerg. Med. J..

[54] Herzig D.A., Brooks R., Mohr C. (2013). Inferring about individual drug and schizotypy effects on cognitive functioning in polydrug using mephedrone users before and after clubbing. Hum. Psychopharmacol..

[55] Grochecki P., Smaga I., Lopatynska-Mazurek M., Gibula-Tarlowska E., Kedzierska E., Listos J., Talarek S., Marszalek-Grabska M., Hubalewska-Mazgaj M., Korga-Plewko A. (2021). Effects of mephedrone and amphetamine exposure during adolescence on spatial memory in adulthood: Behavioral and neurochemical analysis. Int. J. Mol. Sci..

[56] Motbey C.P., Karanges E., Li K.M., Wilkinson S., Winstock A.R., Ramsay J., Hicks C., Kendig M.D., Wyatt N., Callaghan P.D. (2012). Mephedrone in adolescent rats: Residual memory impairment and acute but not lasting 5-HT depletion. PLoS ONE.

[57] López-Arnau R., Martínez-Clemente J., Rodrigo T., Pubill D., Camarasa J., Escubedo E. (2015). Neuronal changes and oxidative stress in adolescent rats after repeated exposure to mephedrone. Toxicol. Appl. Pharmacol..

[58] Krantz M.J., Mehler P.S. (2004). Treating opioid dependence. Growing implications for primary care. Arch. Intern. Med..

[59] Bassetti C.L.A., Kallweit U., Vignatelli L., Plazzi G., Lecendreux M., Baldin E., Dolenc-Groselj L., Jennum P., Khatami R., Manconi M. (2021). European guideline and expert statements on the management of narcolepsy in adults and children. Eur. J. Neurol..

[60] Cortese S., Newcorn J.H., Coghill D. (2021). A practical, evidence-informed approach to managing stimulant-refractory attention deficit hyperactivity disorder (ADHD). CNS Drugs.

[61] Whiting P.F., Wolff R.F., Deshpande S., Di Nisio M., Duffy S., Hernandez A.V., Keurentjes J.C., Lang S., Misso K., Ryder S. (2015). Cannabinoids for medical use: A systematic review and meta-analysis. JAMA.

[62] Friesen C., Hormann I., Roscher M., Fichtner I., Alt A., Hilger R., Debatin K.-M., Miltner E. (2014). Opioid receptor activation triggering downregulation of cAMP improves effectiveness of anti-cancer drugs in treatment of glioblastoma. Cell Cycle.

[63] Lal S., Shekher A., Puneet Narula A.S., Abrahamse H., Gupta S.C. (2021). Cannabis and its constituents for cancer: History, biogenesis, chemistry and pharmacological activities. Pharmacol. Res..

[64] Yoshino A., Ogino A., Yachi K., Ohta T., Fukushima T., Watanabe T., Katayama Y., Okamoto Y., Naruse N., Sano E. (2010). Gene expression profiling predicts response to temozolomide in malignant gliomas. Int. J. Oncol..

[65] Shao H., Chung J., Balaj L., Charest A., Bigner D.D., Carter B.S., Hochberg F.H., Breakefield X.O., Weissleder R., Lee H. (2012). Protein typing of circulating microvesicles allows real-time monitoring of glioblastoma therapy. Nat. Med..

[66] Harrabi S., Combs S.E., Brons S., Haberer T., Debus J., Weber K.J. (2013). Temozolomide in combination with carbon ion or photon irradiation in glioblastoma multiforme cell lines—Does scheduling matter?. Int. J. Radiat. Biol..

[67] Litchfield J.T., Wilcoxon F. (1949). A simplified method of evaluating dose-effect experiments. J. Pharmacol. Exp. Ther..

[68] Jakubowicz-Gil J., Langner E., Badziul D., Wertel I., Rzeski W. (2013). Apoptosis induction in human glioblastoma multiforme T98G cells upon temozolomide and quercetin treatment. Tumor Biol..

[69] Zając A., Sumorek-Wiadro J., Langner E., Wertel I., Maciejczyk A., Pawlikowska-Pawlęga B., Pawelec J., Wasiak M., Hułas-Stasiak M., Bądziul D. (2021). Involvement of PI3K pathway in glioma cell resistance to temozolomide treatment. Int. J. Mol. Sci..

[70] Lemieszek M.K., Nunes F.M., Cardoso C., Marques G., Rzeski W. (2018). Neuroprotective properties of Cantharellus cibarius polysaccharide fractions in different in vitro models of neurodegeneration. Carbohydr. Polym..

[71] Ho J., Tumkaya T., Aryal S., Choi H., Claridge-Chang A. (2019). Moving beyond P values: Data analysis with estimation graphics. Nat. Methods.

