# Supplementary material for: Cell-Specific Vulnerability of Human Glioblastoma and Astrocytoma Cells to Mephedrone—An In Vitro Study
Source: Molecules. 2025 May 22;30(11):2277. doi: 10.3390/molecules30112277 (PMC12156288; doi:10.3390/molecules30112277)
Supplement: Supplementary file 1 [file molecules-30-02277-s001.zip › molecules-3579465-supplementary.pdf]

Supplementary materials

a

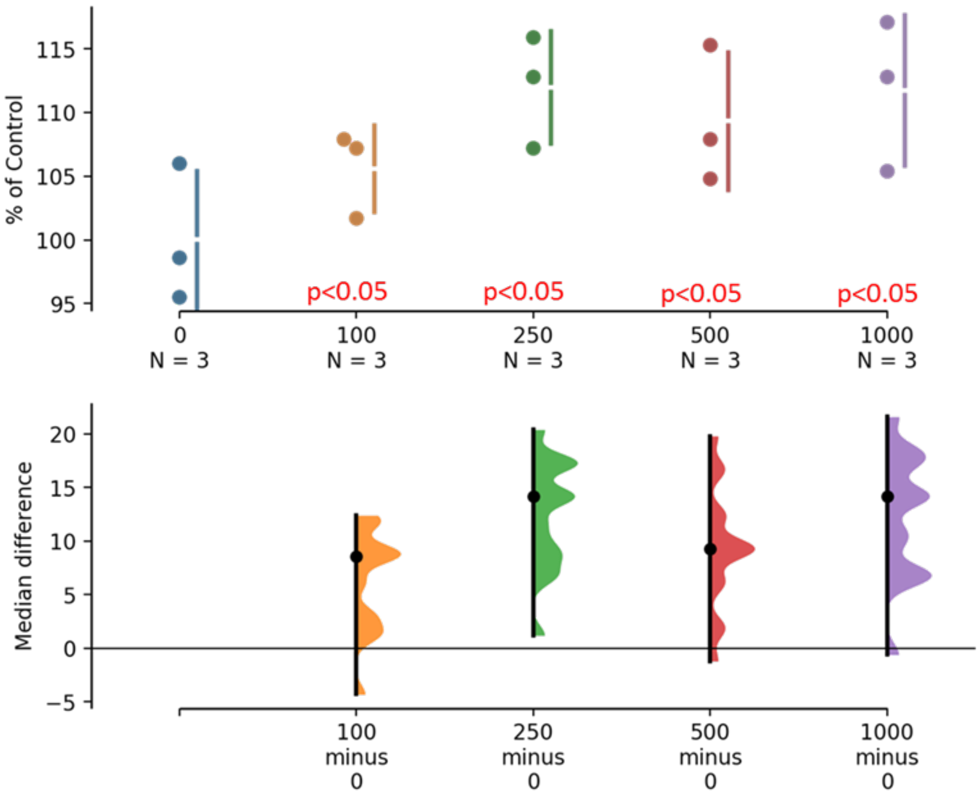

b

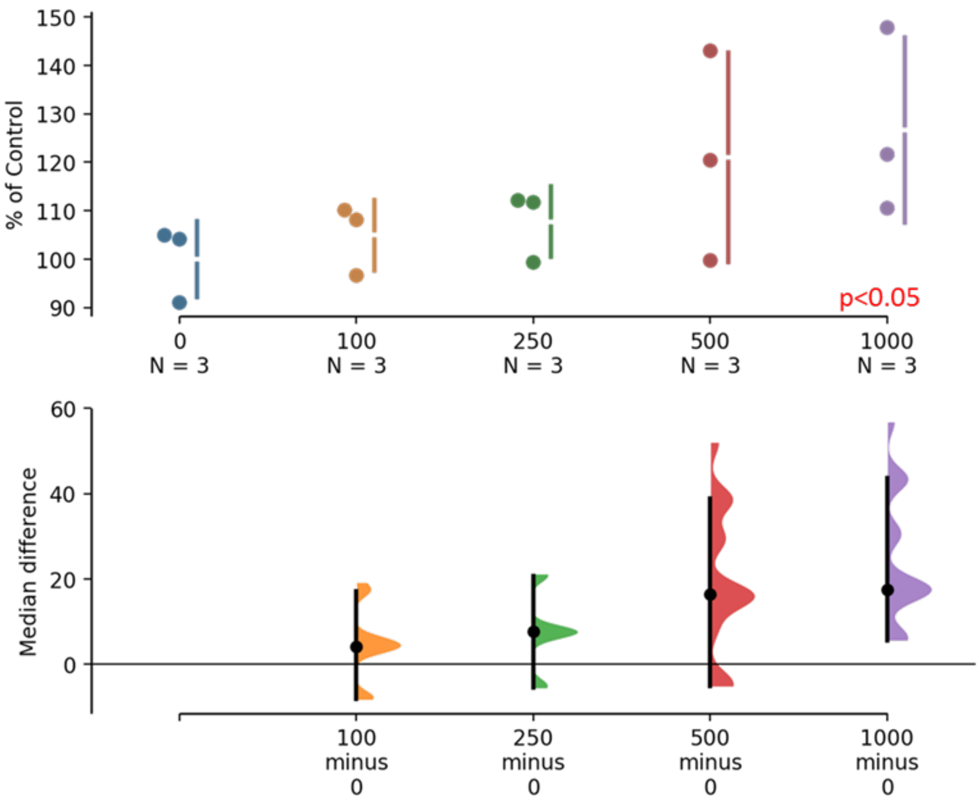

Figure S1. Cytotoxic effect of mephedrone in human glioblastoma LN-18 cells (a) and human glioblastoma multiforme T98G cells (b). Cells were exposed for 48 h to culture medium alone (control) or culture medium containing mephedrone at concentrations 100-1000 µM. Cytotoxic effect was determined by means of the LDH assay of 3 independent trials. The median difference for 4 comparisons against the shared control 0 are shown in the above Cumming estimation plot. The raw data is plotted on the upper axes. On the lower axes, mean differences are plotted as bootstrap sampling distributions. Each mean difference is depicted as a dot. Each 95% confidence interval is indicated by the ends of the vertical error bars. $p<0.05$ vs control was considered significant.

Table S1. The summary of the main genetic and molecular features of investigated cell lines with particular emphasis on alterations associated with cell growth, proliferation, and survival.

		**MOGGCCM**	**LN-18**	**LN-229**	**T98G**
General description [17,18]	Cancer type	human anaplastic astrocytoma	human glioblastoma	human glioblastoma	human glioblastoma multiforme
	Donor gender	unspecified	male	female	male
	Donor age	unspecified	65 years	60 years	61 years
	Donor population	Caucasian	Caucasian	Caucasian	Caucasian
	Stage of diseases	III	IV	IV	IV
Gene *CDKN2A*, which is coding protein p16 (cyclin dependent kinase inhibitor 2A) [19,20]		WT	Homozygous gene deletion; molecular consequences: loss of function.	Homozygous gene deletion; molecular consequences: loss of function.	Homozygous gene deletion; molecular consequences: loss of function.
Gene *TP53*, which is coding tumor suppressor protein p53 [19,20]		Missense mutation (c.476C>T; A159V); affected region: DNA-binding domain; molecular consequences: loss of transcriptional function.	Missense mutation (c.713G>C; C238S); affected region: DNA-binding domain; molecular consequences: loss of transcriptional function.	Missense mutation (c.293C>T; P98L); affected region: proline-rich domain; molecular consequences: impact on protein function unknown .	Missense mutation (c.711G>T; M237I); affected region: DNA-binding domain; molecular consequences: loss of transcriptional function.
Gene *PTEN*, which is coding tumor suppressor protein PTEN (phosphatase and tensin homolog) [19,20]		WT	WT	WT	Missense mutation (c.125T>G; L42R); affected region: phosphatase domain; molecular consequences: loss of function.
Gene *PIK3CB*, which is coding protein PIK3CB (phosphatidylinositol-4,5-bisphosphate 3-kinase		WT	Missense mutation (c.3151G>A; E1051K);	WT	WT

catalytic subunit beta) [19,20]		affected region: catalytic domain; molecular consequences: gain of function.		
Susceptibility to the temozolomide	TMZ-resistant [21,22]	TMZ-resistant [23,24]	TMZ-resistant [25]	TMZ-resistant [23,24]
Methylation of promoter of gene *MGMT* coding enzyme O6-methylguanine-DNA methyltransferase	Unknown	Silenced [26]	Enhanced [26]	Weak [26]
Expression of MGMT	Unknown	Up-regulated [26]	Down-regulated [26]	Up-regulated [26]
IDH1 (isocitrate dehydrogenase 1)	Unknown	WT [27]	WT [27]	WT [27]

Table S2. IC_{50} values of mephedrone were calculated for selected human glioblastoma and astrocytoma cell lines as well as rat oligodendrocytes based on results of MTT assays performed after 96 h of cell treatment. IC_{50} value (concentration causing proliferation inhibition by 50% compared to the control) was calculated according to the Litchfield and Wilcoxon method using GraphPad Prism 5. $^{*}IC_{50}$ value was calculated based on the previously published results [15].

Cell line	Histotype	IC_{50} (μM)	Confidence limits (μM)
LN-18	Glioblastoma	1728	1337-2235
LN-229	Glioblastoma	673	555-816
T98G	Glioblastoma multiforme	277	254-301
MOGGCCM	Anaplastic astrocytoma	853	715-1017
OLN-93*	Oligodendrocytes	612	581-644

Table S3. Percentage of cells displaying apoptosis, necrosis and autophagy after 24 and 48 h incubation in the presence of mephedrone. Data are presented as a percentage of cells in which specific type of cell death was observed. Mephedrone 0 μM means control. At least 1000 cells in randomly selected microscopic fields were counted under the microscope. Each experiment was conducted independently in triplicate. Statistical analysis was performed using one way-ANOVA with the Dunnett's *post hoc* test. $^{*}p<0.05$ vs control was considered significant.

Cell line	Cell death type	Mephedrone [μM]									
		0	100	250	500	1000	0	100	250	500	1000
		Incubation time 24 h					Incubation time 48 h				
LN-18	apoptosis	0.1	0.7	1	7*	5	0.1	0.2	2.5	4.5	4.9
	necrosis	0	0	0	0	3	0	0	0	0	0.8
	autophagy	0	0	0.2	1	1	0	0	2	1.3	0.9
LN-229	apoptosis	0.5	5	6.4*	7.9*	8*	0.7	1.9	2.9	6.8*	7.2*
	necrosis	0	0	0	0	0	0	0	0	0	0
	autophagy	0	0	0	0.5	3	0	0	0	1.7	3.1
T98G	apoptosis	0	0	0	3.4	4	0.1	0	2.5	4.5	5.1
	necrosis	0	0	0	0	0	0	0	0	0	0
	autophagy	0	0	0	0	0	0	0	0	0	0.9
MOGGCCM	apoptosis	0	0	0	0	2	0	0	1	2	3
	necrosis	0	0	0	0	0	0	0	0	0	0
	autophagy	0	0	0	0	0	0	0	0	0	0.9

Table S4. The concentration (µM) of temozolomide and mephedrone in gliomas cell lines.

Gliomas cell line	**Temozolomide** IC_{50} [µM]			**Mephedrone** IC_{50} [µM]
	[64]	[65]	[66]	manuscript by Marszalek-Grabska (under review)
A-172	52	-	-	-
AM-38	41	-	-	-
T98G	442	200	-	277
U-87MG	23	-	-	-
U-138MG	387	-	-	-
U-251MG	22	-	-	-
YH-13	371	-	-	-
SF295	-	80	-	-
LN-18	-	-	560	1728
LN-229	-	-	5	673
MOGGCCM	-	-	-	853
